# Creatures of habit (and control): a multi-level learning perspective on the modulation of congruency effects

**DOI:** 10.3389/fpsyg.2014.01247

**Published:** 2014-11-06

**Authors:** Tobias Egner

**Affiliations:** Center for Cognitive Neuroscience, Department of Psychology and Neuroscience, Duke University, Durham, NC, USA

**Keywords:** cognitive control, feature integration, memory, attention, congruency sequence effect, proportion congruent effect, conflict adaptation, contingency learning

## Abstract

The congruency sequence effect (CSE) describes the finding that congruency effects in classic probes of selective attention (like the Stroop, Simon, and flanker tasks) are smaller following an incongruent than following a congruent trial. The past two decades have generated a large literature on determinants and boundary conditions for the CSE and similar, congruency-proportion based modulations of congruency effects. A prolonged and heated theoretical discussion has been guided primarily by a historically motivated dichotomy between “top-down control” versus “associative bottom-up” explanations for these effects. In the present article, I attempt to integrate and contextualize the major empirical findings in this field by arguing that CSEs (and related effects) are best understood as reflecting a composite of multiple levels of learning that differ in their level of abstraction. Specifically, learning does not only involve the trial-by-trial encoding, binding, and cued retrieval of specific stimulus–response associations, but also of more abstract trial features. Moreover, these more abstract trial or event features can be both external, such as the spatial and temporal context in which a stimulus occurs, as well as internal, like the experience of difficulty, and the attentional control settings that were employed in dealing with the stimulus. From this perspective, top-down control and bottom-up priming processes work in concert rather than in opposition. They represent different levels of abstraction in the same learning scheme and they serve a single, common goal: forming memory ensembles that will facilitate fast and appropriate responding to recurring stimuli or events in the environment.

## INTRODUCTION

**FIGURE 1:**
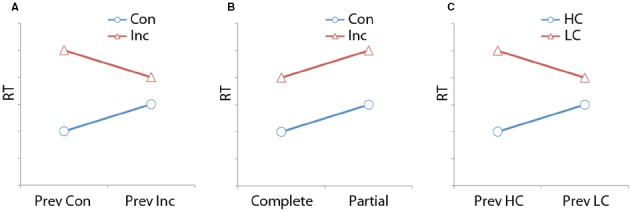
**Different interpretations of first-order sequence effects in conflict tasks. (A)** Hypothetical data plotted and labeled as a congruency sequence effect (CSE), where response time (RT) varies as a function of an interaction between previous trial congruency and current trial congruency. Con, congruent; Inc, incongruent; Prev, previous trial. **(B)** The same data as in **(A)**, plotted in line with the feature integration account, whereby the data pattern represents two additive effects of congruency and feature repetitions. Complete, complete feature repetitions or complete feature alternations from previous trial; Partial, partial feature repetitions from previous trial. **(C)** The same data and plotting as in **(A)**, but relabeled in line with the interpretation of the contingency learning account, where the data are explained as an interaction between previous trial and current trial contingency effects. LC, low-contingency trials; HC, high-contingency trials.

Tests of the effectiveness of controlled attention typically require participants to produce a response to a task-relevant stimulus feature (target information) in the presence of task-irrelevant stimulus features (distracter information), which can be either congruent or incongruent with the former. For instance, in the classic color-naming Stroop task (Stroop, 1935; MacLeod, 1991), subjects have to indicate the ink color of written color-words (e.g., RED) while ignoring the word-meaning. The relative success (or failure) of attentional filtering is gauged by contrasting performance on trials where the distracter is congruent (e.g., the word RED written in red ink) with those where it is incongruent (e.g., the word RED written in blue ink) with the target, and may therefore interfere with target processing unless it is effectively ignored. The canonical finding is a marked congruency effect: responses are slower and more error-prone to incongruent compared to congruent stimuli, suggesting imperfect attentional selection. Importantly, the size of the congruency effect, and by implication, the effectiveness of attentional filtering, has been shown to be malleable by a variety of factors, such as the frequency of incongruent stimulus occurrences (Logan and Zbrodoff, 1979), the explicit cueing of forthcoming congruency (Gratton et al., 1992), and the congruency of the previous trial (Gratton et al., 1992). The latter refers to the so-called *congruency sequence effect* (CSE), the finding that the influence of distracters on the processing of target information is typically dampened on trials that follow an incongruent trial compared to those that follow a congruent trial (Figure 1A; for reviews, see Egner, 2007; Duthoo et al., 2014a).

Over the past two decades, the CSE has garnered much attention in the Cognitive Psychology and Neuroscience literatures, with a central debate focusing on rival explanations for this phenomenon, which are typically grouped into two major categories, “top-down control-based” versus “bottom-up associative” accounts (see, e.g., Gratton et al., 1992; Botvinick et al., 2001; Mayr et al., 2003; Hommel et al., 2004; Blais et al., 2007; Egner, 2008; Verguts and Notebaert, 2008, 2009; Hazeltine et al., 2011; Schmidt, 2013). In the present hypothesis article, I attempt to provide an integrative perspective on these accounts, as well as related phenomena of contextual modulations of congruency effects. Put simply, I will argue that the different accounts of these effects’ origins ultimately describe complementary learning processes operating at different levels of abstraction but driven by a common principle and goal: the matching of incoming stimulation (external states) to memories (internal states) in the service of producing fast, goal-conducive action. At the most concrete level, the organism binds together co-occurring physical stimulus and response features, whereas at a more abstract level, we associate complex contextual cues with generalizable control states. I will first provide a brief overview of some rival CSE accounts and the empirical status quo, followed by the main argument for viewing distinct sources of CSEs as describing different, co-occurring levels of a broader learning process aimed at optimizing stimulus processing and action selection.

## A SELECTIVE COMPENDIUM OF CSE ACCOUNTS

### CONTROL-BASED PERSPECTIVES

In line with the standard interpretation of congruency effects as reflecting a measure of attentional selectivity, the original observation of the CSE was interpreted as the expression of an adjustment in attentional strategy (Gratton et al., 1992). More specifically, Gratton et al. (1992) reasoned that encountering a congruent or incongruent trial would engender an expectation for the forthcoming trial to be of the same congruency (cf. Remington, 1969), which in turn would lead subjects to strategically enhance (following incongruent trials) or decrease (following congruent trials) their attentional focus on the target stimulus feature, thus decreasing the influence of distracters (and, *ergo*, the congruency effect) following an incongruent trial, and increasing the influence of distracters (and, *ergo*, the congruency effect) following a congruent trial.

About a decade later, a related, though much more formalized (and influential), control-based account for the CSE was put forward by Botvinick et al. (2001), who marshaled this effect as evidence to support a “conflict-monitoring” model of cognitive control. Briefly, these authors advanced an elegant computational scheme for a “homunculus-free” regulation of top-down attention, positing that the cognitive apparatus detects internal conflict between mutually incompatible stimulus or response representations (e.g., the simultaneous urge to answer “red” and “blue” when faced with the incongruent Stroop stimulus described above), and uses the degree of conflict to produce commensurate adjustments in top-down attention—the more conflict is experienced, the more control will be applied. Thus, when an incongruent stimulus is encountered, the processing conflict caused by the incongruent distracters triggers an up-regulation of attentional focus toward the target, which results in more efficient attentional selection (and hence, a smaller congruency effect) on the following trial; the opposite is true when encountering a low-conflict, congruent trial, which results in a relaxation of attention and, thus, less efficient attentional selection (and a larger congruency effect) on the following trial. This particular interpretation of the CSE is known as *conflict adaptation*.

While there are clear conceptual differences between the expectation- and conflict-based accounts (see, e.g., Egner, 2007; Egner et al., 2010; Duthoo et al., 2013; Jimenez and Mendez, 2013), for the present purpose they can both be considered core members of the “control-based” model category, along with various proposed refinements and extensions of the basic conflict-monitoring proposal (e.g., Botvinick, 2007; Egner, 2008; Hazeltine et al., 2011; Dreisbach and Fischer, 2012; Jiang et al., 2014). First, these views commonly assume that the CSE results from strategic adjustments in top-down attention or task-set. Second, and more importantly, these accounts all operate at a level of processing adjustment that is, in principle, independent of what the specific stimulus features or responses are that will comprise the subsequent trial. For instance, conflict- (or expectation-) triggered enhanced attentional filtering in the Stroop task would result in improved ink color selection regardless of the exact nature of that color or the distracter word information in the upcoming trial. As will be discussed in more detail later on, this “level of abstraction” of the mechanism that is held responsible for the CSE, concerning either a *generalizable cognitive state* (for instance, attentional focus) or *specific stimulus or response characteristics*, represents the key distinguishing feature between control-based and associative accounts of the CSE.

According to this criterion, we can also subsume under the control-based category CSE accounts that focus on control adjustments at the level of response selection rather than perceptual attention. For instance, the “activation–suppression model” posits a control mechanism that detects and suppresses response activation elicited by distracter stimuli, and assumes this mechanism to work more effectively if it had been recently activated (i.e., by a prior incongruent trial; e.g., Ridderinkhof, 2002; van den Wildenberg et al., 2010). Crucially, this model nevertheless assumes that the process which is facilitated following an encounter with an incongruent stimulus, namely the categorical suppression of “distracter-route” responses, is independent of the specific features of the subsequent stimulus. By contrast, certain “hybrid accounts” that espouse the notion of conflict-enhanced control, but link this mechanism to specific stimulus features (Blais et al., 2007; Verguts and Notebaert, 2008; Blais and Verguts, 2012), defy the present categorization scheme; these models will be discussed in Section “A Multi-level Learning Perspective on the Modulation of Congruency Effects.”

### ASSOCIATIVE PERSPECTIVES

Associative accounts have proposed that the CSE may stem from memory-driven effects, based on differing frequencies with which specific stimulus and response features repeat over consecutive trials for different congruency sequences (Mayr et al., 2003; Hommel et al., 2004). For instance, Hommel et al.’s (2004) *feature integration* account is grounded in prior work showing that the specific stimulus and response features that co-occur on a given trial of an alternative forced-choice (AFC) task (say, the word RED in blue ink is responded to with a left button press in the above-mentioned Stroop task) become *bound together* in episodic memory as an “event file” (Hommel, 1998, 2004; cf. Treisman and Gelade, 1980). Moreover, the subsequent re-occurrence of any one of these features (e.g., the word RED) appears to trigger the retrieval of the entire prior event file, presumably to supply a potential shortcut to the correct response associated with a previously seen stimulus (cf. Logan, 1988). This feature-binding mechanism leads to a relative facilitation of processing when all of the current trial features match the previous event (*complete repetitions*; see also Pashler and Baylis, 1991; Mayr et al., 2003), or when there is no feature overlap across successive trials (*complete alternations*), relative to cases where some features are repeated but others are not (*partial repetitions*), because in the latter scenario, the retrieved event file has to be either discarded or “unbound” in order for the currently presented stimulus to be responded to correctly (Hommel, 1998, 2004; see also Neill, 1997).

By way of example, consider once more the Stroop task alluded to earlier, consisting of a stimulus set of the words RED and BLUE, printed in either red or blue ink, thus rendering a total of two congruent and incongruent stimuli, and two possible responses. Here, congruent–congruent and incongruent–incongruent trial sequences will consist entirely of complete feature repetitions or complete alternations (and thus, result in fast responses), whereas congruent–incongruent and incongruent–congruent trial sequences will consist entirely of partial feature repetitions (thus resulting in slow responses). Hence, the CSE data pattern of reduced congruency effects following an incongruent trial compared to a congruent trial can be re-interpreted as reflecting a basic congruency effect paired with a relative handicapping of trials where partial feature repetitions impose an “unbinding cost” on performance (Figure 1B; Hommel et al., 2004). Evidently, in contrast to the control-based accounts, this associative perspective requires no trial-by-trial adjustments of selective attention to explain the CSE, and, importantly, the proposed mechanism underlying this effect operates at the level of specific stimulus features and responses.

Given that the feature integration account highlights potential associative confounds in the CSE that seem to be specifically inherent to small stimulus and response sets, a natural response to these concerns was a movement toward employing conflict tasks with larger sets (typically, moving from 2-AFC to 4-AFC schemes), such that first-order repetitions of stimulus and response features could be either prophylactically prevented from occurring (e.g., Puccioni and Vallesi, 2012; Jimenez and Mendez, 2013), or removed from analysis after the fact (e.g., Ullsperger et al., 2005; Akcay and Hazeltine, 2007). However, as recently highlighted by several authors (Schmidt and De Houwer, 2011; Mordkoff, 2012), this trend may have introduced a new associative confound to the CSE, in the form of *contingency learning*. Specifically, the expansion of the stimulus set (for instance, going from two to four colors in the Stroop task) creates more possible unique incongruent than congruent stimuli. When researchers then present congruent and incongruent trials with the same frequency (i.e., 50%), each congruent stimulus occurs more frequently (and well above chance) than each incongruent stimulus, which creates a contingency linking each distracter to their congruent response (e.g., the word RED is most frequently paired with the color red, and thus, the response “red”). Since high-contingency (congruent) trials are responded to faster than low-contingency (incongruent) trials, *and* consecutive trials with the same contingency level appear to facilitate performance (Schmidt and De Houwer, 2011), it is possible that the CSE in typical 4-AFC tasks is a reflection of contingency-learning rather than of control-based processing adjustments (Figure 1C).

At this point, it is worth to already highlight an overarching commonality between the “control-based” and “associative” mechanisms that have been proposed as explanations of the CSE, namely that they share the same ultimate purpose: the reason for (i.e., the evolutionary selection for) binding together stimulus features and actions is of course that this will facilitate fast and appropriate responding to recurring stimuli (in a world where recurring stimuli and events are the norm). In other words, the organism creates memories and tries to match those memories to external stimulation, such that previously experienced events do not have to be processed “from scratch” like novel events. The same basic purpose, but at a more abstract level, is served by the putative control-based mechanisms noted above; they all describe an adaptation of processing strategies to previously experienced (and likely recurring) events, which serves the goal of being prepared for similar challenges in the future—another instance of matching memories (here, of control states) to external stimulation in order to facilitate fast and accurate responses.

## THE EMPIRICAL STATUS QUO

The empirical evaluation of control-based and associative sources of the CSE has produced a substantial literature over the last decade, the nuances of which are discussed elsewhere in much greater detail than I aim to provide here (e.g., Egner, 2007; Hazeltine et al., 2011; Schmidt, 2013; Duthoo et al., 2014a; Weissman et al., 2014); instead, the present section summarizes what I consider to be the key take-home messages of that literature. First, there is little doubt that the nature of overlap in stimulus and response features over successive trials can profoundly affect performance (Hommel, 1998) and it is *impossible* to circumvent the confounding factor of differential feature overlap between different congruency sequences in the CSE when employing small stimulus sets (e.g., only two or three different target and distracter stimuli). It is therefore possible, or even likely, that CSEs observed in studies with such small stimulus sets are partly, predominantly, or entirely driven by feature integration effects (e.g., Mayr et al., 2003; Hommel et al., 2004; Nieuwenhuis et al., 2006; Notebaert et al., 2006).

Second, while the movement toward employing larger stimulus sets has resulted in a number of studies reporting CSEs in the absence of feature repetitions (e.g., Ullsperger et al., 2005; Akcay and Hazeltine, 2007, 2011; Hazeltine et al., 2011), almost all of these studies appear to be open to alternative interpretation based on possible contingency-learning confounds because of above-chance occurrence of congruent stimuli (see Schmidt, 2013). Similarly, 2-AFC studies that require subjects to categorize large sets of unique stimuli (e.g., classifying face stimuli according to gender) have produced CSEs in the absence of any stimulus feature repetitions (e.g., Egner and Hirsch, 2005; Egner et al., 2008, 2010; Lee and Cho, 2013), but they have been criticized as being vulnerable to possible feature integration effects operating at the level of semantic categories (like “male” and “female”) rather than specific stimulus features (Schmidt, 2013; but see Jiang et al., 2014).

Third, however, a substantial crop of recent papers with designs that specifically control for both feature integration *and* contingency learning confounds have in fact reported robust CSEs (Kunde and Wuhr, 2006; Freitas and Clark, 2014; Hengstler et al., 2014; Kim and Cho, 2014; Schmidt and Weissman, 2014; Weissman et al., 2014; but see Mayr et al., 2003; Jimenez and Mendez, 2013). A typical design of this recent wave of studies circumvents both stimulus and response feature repetitions, as well as contingency-learning confounds, by splitting a 4-AFC task into two alternating 2-AFC tasks with non-overlapping stimulus and response sets (e.g., presenting alternately Stroop stimuli that are made up either of red/blue or of green/yellow combinations; e.g., Schmidt and Weissman, 2014). This approach has produced robust evidence for the basic presence of a “memory confound-free” CSE, though in and of itself this does of course not tell us what other, possibly control-based, mechanism is mediating these effects. Current studies are starting to address this question by exploring the precise boundary conditions for obtaining a CSE under these constraints (e.g., Kim and Cho, 2014; Weissman et al., 2014).

For the present purpose, the key conclusion is that there is solid evidence that CSEs can be produced both by sources that operate at a *feature-specific* level, driven by (re-)occurrences of particular physical stimulus and response characteristics, as well as by sources that must operate at a more *abstract* level, producing CSEs in a manner that is independent of repetitions of specific stimulus and response characteristics. How these distinct contributors to the CSE may be conceptualized most fruitfully within a single framework is the subject of the following section.

## A MULTI-LEVEL LEARNING PERSPECTIVE ON THE MODULATION OF CONGRUENCY EFFECTS

### MEMORY (OBVIOUSLY) FORMS THE BASIS OF ALL SEQUENCE EFFECTS

First, it is worth emphasizing a point that perhaps seems self-evident, but which has often been lost sight of in the long-running debates over different possible causes for the CSE. That point is that *any* time that a past experience (e.g., encountering an incongruent trial) affects current performance, we are, by definition, dealing with an expression of *memory* (or *learning*). This is true regardless of whether the effect be mediated by some kind of “passive carry-over” of within-trial conflict-resolution dynamics (see, e.g., Egner et al., 2010; van den Wildenberg et al., 2012), or whether the prior trial served to explicitly engender expectations regarding the forthcoming congruency level (e.g., Gratton et al., 1992), or any other previously articulated “control-based” mechanism; at the end of the day, these are all means by which past experience changes current behavior, and are thus instances of memory. Unsurprisingly, therefore, whenever researchers have gone to the effort of constructing formal computational models of the CSE, these were grounded in a *reinforcement learning* algorithm (Botvinick et al., 2001; Blais et al., 2007; Verguts and Notebaert, 2008), where experienced conflict essentially acts as a teaching signal for updating the manner in which forthcoming stimuli will be processed (cf. Jiang et al., 2014). Hence, the basic dichotomy implied in labeling CSE accounts “control-” or “attention-based” versus “associative” or “memory-based” can be misleading. Rather than asking, “is it memory or is it attention?” we must ask: “what *type of learning processes* contribute to the CSE?”

**FIGURE 2:**
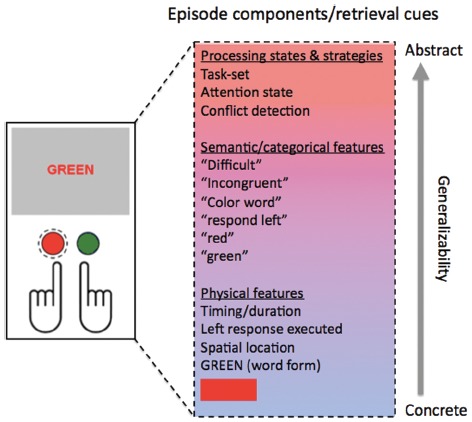
**Concrete and abstract features associated with an event or episode file pertaining to a trial on the color-naming Stroop task.** The features portrayed here (and possible additional ones) are proposed to be bound together into an “episode file” that will be retrieved when cued by matching features in future trials on the task.

To this end, as has already been alluded to in previous sections, I submit that a useful re-conceptualization of the traditional dichotomy between control-based and associative accounts should not focus on the juxtaposition of attention versus memory, but on the *level of abstraction* at which memory or learning effects are being expressed. The feature-integration and contingency-learning accounts deal with learning that links together *concrete* trial characteristics, namely, particular stimulus features (and perhaps categories) and responses; by contrast, the control-based accounts are concerned with learning aspects of a trial that transcend the physical specifics of the stimuli or responses, dealing instead with more *abstract* properties, like congruency, experienced conflict, and/or the cognitive mechanisms that were recruited for dealing with the latter (Figure 2). As concluded in the brief review of the empirical literature above, there is little doubt that both concrete- and abstract-level learning processes take place and that they are each able to produce CSEs in their own right (as well as presumably contributing to CSEs simultaneously when tasks are not designed to isolate them). Two pertinent questions, then, are what exactly the abstract trial characteristics might be that are being learned, and how we should best conceptualize the relationship between the different levels of learning.

### WHAT KIND OF LEARNING MEDIATES CONCRETE-FEATURE INDEPENDENT CSES?

The “associative” accounts of the CSE explain this effect as a consequence of the binding between concrete stimulus features and responses, which shapes the future processing of similar events. In the absence of concrete feature overlap or biased stimulus–response contingencies, what is it that might be learned or remembered from trial-to-trial that would produce a “control-based” CSE? While the answer is presently not certain, the empirical literature allows us to impose some bounds on possible candidates. First, we can likely reject the notion of a very general effect, whereby exposure to an incongruent trial would lead to broad performance benefits regardless of the specifics of the task-demands on the forthcoming trial. This rejection is based on a large number of studies that have documented the CSE to be *domain-* or *conflict-specific* (e.g., Wendt et al., 2006; Egner et al., 2007; Boy et al., 2010; Funes et al., 2010; Akcay and Hazeltine, 2011; Schlaghecken et al., 2011; Kim et al., 2012; Kunde et al., 2012; for reviews, see Egner, 2008; Braem et al., 2014). Such conflict-specificity is most appropriately assessed in protocols that combine factorially two distinct types of conflict into a single task, such that the independence of conflicts (i.e., additive main effects) and their potential sequential (in)-dependence can be assessed simultaneously, and in the absence of potential task-switching effects when alternating between conflicts in a non-factorial design (for an extended discussion, see Egner, 2008). For example, if one combines the Stroop and Simon tasks, by presenting color-words to the left or right of a central fixation and requiring subjects to respond to ink color using left and right response buttons, one obtains additive Simon and Stroop congruency effects (Simon and Berbaum, 1990; Kornblum, 1994; Jiang and Egner, 2014) and conflict-specific CSEs: Stroop congruency effects are reduced following a Stroop-incongruent stimulus, but not following a Simon-incongruent stimulus, and *vice versa* (Egner et al., 2007). As concluded in a recent, more extensive review of this topic, in studies using designs that assessed the conflict-specificity of CSEs in the absence of potentially confounding task-switch effects (cf. Egner, 2008) there is abundant evidence for such specificity (Braem et al., 2014). Thus, much evidence suggests that abstract learning contributions to the CSE must operate at a relatively “local,” trial type-specific level rather than at a global, highly generalizable level.

In this vein, two closely related “hybrid” models of the CSE, alluded to earlier, have argued for a very local, *stimulus-specific* learning process (Blais et al., 2007; Verguts and Notebaert, 2008; see also Blais and Verguts, 2012). For instance, the “adaptation-by-binding” model proposes that the occurrence of conflict triggers an arousal response that enhances the association between top-down attention (task demand units) and the currently activated input units, which would correspond to the *specific stimulus features* of the present incongruent stimulus (Verguts and Notebaert, 2008, 2009). The authors propose that this form of “conflict-modulated Hebbian learning,” binding top-down attention to conflict-evoking stimuli^1^, can account both for the CSE and a related phenomenon called the “item-specific proportion congruent” (ISPC) effect (Jacoby et al., 2003), where congruency effects are selectively reduced for specific task-irrelevant stimulus features (e.g., the word RED) that are frequently presented as part of incongruent stimuli. The adaptation-by-binding model can indeed easily explain the ISPC effect as reflecting repeated strengthening of connections between task demand units and specific stimulus features (see also Blais et al., 2007). Explaining the (non-specific) CSE, by contrast, is attributed to the model assumption that input units for task-relevant features which are not actually part of the current stimulus are also “slightly activated,” such that even non-present stimulus features are held to be subject to conflict-modulated Hebbian learning (Verguts and Notebaert, 2008).

However, this stimulus-specific learning approach appears to be incapable of explaining conflict-specific CSEs in tasks where two conflict types are combined in a factorial design, such that the basic stimulus features do not actually differ between conflict types (see Egner, 2008). Here, the adaptation-by-binding model would predict generalizable benefits of conflict-enhanced binding of attention to (especially task-relevant) stimulus features, regardless of which type of conflict elicited the putative arousal response and binding process; this runs counter to empirical findings, however, because even with identical task-relevant features (e.g., ink color), conflict-general CSEs are not observed in such studies (Wendt et al., 2006; Egner et al., 2007; Boy et al., 2010; Akcay and Hazeltine, 2011; Schlaghecken et al., 2011; Kim et al., 2012; Kunde et al., 2012). There are some studies showing CSEs to selectively cross “conflict boundaries” when task-relevant features are shared rather than distinct (e.g., Notebaert and Verguts, 2008), but in these studies, changes between conflict types also represent switches between tasks, such that they cannot directly speak to the conflict-specificity (or lack thereof) of CSEs (see Egner, 2008). These types of findings do suggest, however, that rendering different tasks more similar may promote the likelihood of obtaining cross-task CSEs (Akcay and Hazeltine, 2008; for additional discussion, see Braem et al., 2014). To return to the adaptation-by-binding model though, by conceptualizing conflict as strengthening attentional modulation of *specific input units* (e.g., “red”; Verguts and Notebaert, 2008) rather than of the general task-relevant processing pathway (e.g., “attend to color”), it is also difficult to see how this item-based account can capture other, non-specific effects, such as effects of proportion congruency on completely novel or “unbiased” stimuli, which have been demonstrated both in the domain of the ISPC effect (Bugg et al., 2011) as well as in the related domains of “list-wide” (Bugg and Chanani, 2011) and “context-specific” proportion congruent (CSPC) effects (Crump et al., 2006; Crump and Milliken, 2009; Heinemann et al., 2009; King et al., 2012), which are addressed in more detail below. In sum, while I am sympathetic to the idea of a learning process that directly associates top-down states with bottom-up trial features (see below), accounts that focus on binding attention only to *concrete* stimulus features (Blais et al., 2007; Verguts and Notebaert, 2008) appear to be too narrow to explain a wide array of relevant findings.

From this discussion, we can conclude that the level of (abstract) learning that might mediate CSEs in the absence of concrete feature memory effects can neither be so broad as to cross conflict or task boundaries, nor so narrow as to prevent generalization to novel or unbiased stimuli within the same task or conflict type. As elaborated in the following sections, a parsimonious level of learning therefore would consist of the binding of task- (or conflict-) specific but feature-independent top-down states (e.g., “attend to color,” or “suppress responses to location”) to contextual cues, which can range from specific stimulus features to temporal episodes.

### EVENT FILES THAT BIND CONTROL TO CONTEXT

In order to appreciate the level of abstraction that in my view best captures the type of learning that contributes to feature-independent CSEs (and a range of similar effects), consider the phenomenon of the CSPC effect (for a recent review, see Bugg and Crump, 2012): for example, Crump and Milliken (2009) displayed Stroop stimuli in two different locations, either at the top or bottom of the screen, and unbeknownst to the subjects, the likelihood of incongruent stimuli was high at one location and low at the other (but 0.5 overall). Under this set-up, subjects displayed smaller congruency effects in the location with a high incidence of incongruent trials, suggesting they had learned (implicitly, as it turns out) to associate the two spatial contexts with different attentional requirements or settings. Most importantly, this effect held even for unbiased “transfer” stimuli that had occurred with equal frequency at the two locations. These and similar findings in the context of the ISPC (Bugg et al., 2011), CSPC (Heinemann et al., 2009; King et al., 2012), and “list-wide” proportion congruent effects (Bugg and Chanani, 2011), are all pointing toward the same conclusion: that *generalizable* (i.e., abstract) control states can be bound to contextual cues (location, in the above example). I argue here that the “control-based” CSE represents but one particular instance of the workings of this fundamental associative mechanism that binds external cues to internal states.

One simple and parsimonious way of thinking about the more abstract levels of learning mediating CSEs, therefore, is to extend the idea of event files to encompass not just the forming of associations between concrete stimulus and response features, but also of more abstract, categorical stimulus features and, importantly, the linking of these features with co-occurring internal cognitive states, most pertinently, with the *attentional* or *control state* that was being engaged during the processing of said stimulus event (Figure 2). Note that while that control state may in fact be elicited by, and serve the resolution of, “conflict,” the idea that internal control settings are being bound to contextual cues is not in any way dependent on conflict being a driving factor; it could equally well be another representation of task difficulty or task requirements that is being bound to, and retrieved in response to, a contextual cue (e.g., Crump and Logan, 2010). In either case, a subsequent cued retrieval of that event file, for example, by one of the stimulus features, would not just prime other associated physical stimulus and response features, but also the retrieval of the associated top-down attentional set which, given its abstract nature (for instance, “highly focused attention on ink color”), can be generalized to novel or unbiased “transfer” items (for similar arguments, see Spape and Hommel, 2008; Crump and Logan, 2010; Hazeltine et al., 2011; King et al., 2012). At the same time, as noted in the discussion of conflict- and task-specificity of CSEs above, the control states that are being incorporated into the event file appear to be constrained in their generality, in that they reflect the particular settings of the current task-set or control process. Accordingly, for example, contextual cueing of the top-down process involved in resolving Stroop conflict, thought to involve primarily the biasing of stimulus processing stages (Kornblum et al., 1990; Egner and Hirsch, 2005; Egner et al., 2007), will be of little use when faced with Simon conflict, which is thought to be incurred and resolved at the response-selection stage (Kornblum et al., 1990; Sturmer et al., 2002; Sturmer and Leuthold, 2003; Egner et al., 2007; Shin et al., 2010), and *vice versa*.

### TEMPORAL CONTEXT: MOVING FROM EVENTS TO EPISODES

It is easy to appreciate how this kind of mechanism could account for “unbiased” ISPC and CSPC effects (e.g., Crump and Milliken, 2009; Bugg et al., 2011), where a stimulus or contextual feature, over repeated pairings, becomes associated with a task-appropriate control state. It is less obvious, however, how such context-control binding mechanism would account for a CSE in the absence of any concrete feature overlap (and thus, bottom-up cue) across trials. What if there were no such matching cue or, put another way, what would lead to an appropriate and generalizable control state being engaged *in anticipation* of the arrival of a stimulus? The answer brings us all the way back to the notion of *expectations* or *predictions* which are inherent in both the Gratton et al. (1992) and the Botvinick et al. (2001) explanations of the CSE, but I will here attempt to integrate these ideas within the same framework as the event files: specifically, in order to explain *generalizable* control effects at the trial-by-trial level in the absence of concrete feature repetition, a second conceptual expansion of the event file scheme is required. In particular, while “context” in the empirical studies cited above has been operationalized in the limited terms of concrete stimulus features, such as stimulus location or color, it is fruitful to abstract this notion further, so as to include the concept of a *temporal context*, meaning that the retrieval or priming of particular stimulus–response links and/or control states can be based on a temporally defined frame the organism believes itself to be in (e.g., Braver and Barch, 2002; Koechlin et al., 2003). This suggestion essentially equates to extending the discrete and instantaneous nature of an “event file” to a more dynamic and extended form of an “episode file,” which can encode and (upon retrieval) apply temporally extended contingencies and task sets (akin to *schemas*). This broader conception of context would allow for stimuli in the spatial and/or temporal vicinity of the current focus of processing to guide appropriate event/episode file retrieval.

Temporal context can be relatively local (informed primarily by the most recent events) or global (based on a longer sequence of events). In a laboratory setting, a *global* temporal contextual cue would correspond to a particular task phase or block of trials that is predictive of concrete (e.g., color) or abstract (e.g., congruency) stimulus characteristics; “list-wide” proportion congruent effects are one obvious case in point (e.g., Bugg and Chanani, 2011). In real life, episodic frames (e.g., “am I at my own house or at my grandmother’s place?”) routinely determine the retrieval of extended schemas that include appropriate control states (cf. Miller and Cohen, 2001; Koechlin and Summerfield, 2007). By contrast, the most *local* contextual cue corresponds to the most recently sampled observation (i.e., the previous trial), which serves to update the organism’s running estimate of the likely nature of forthcoming events (e.g., Sutton and Barto, 1998). Thus, in the context of the CSE, encountering an incongruent trial sets a context under which the subjective likelihood of a forthcoming trial also being incongruent is enhanced, as is inherent in the accounts of Gratton et al. (1992) and Botvinick et al. (2001). Importantly, the degree to which a given event drives the updating of the organism’s belief of the nature of its current temporal context is conditional on the statistics of the environment. In a fast-changing (volatile) environment, local temporal context (e.g., the last trial) should be more influential, whereas in a more stationary (stable) environment, a more global temporal context (e.g., a block of trials) provides a more reliable cue for forthcoming stimulation (Behrens et al., 2007). In line with this proposition, it was recently shown that a model using a volatility-modulated learning rate in its prediction of forthcoming task demands could capture both the CSE as well as a simultaneous proportion congruent effect^2^ in a Stroop-like task (Jiang et al., 2014).

According to the current proposal, the local temporal context will prime (or maintain) the activation of the control state (as well as the lower-level stimulus and response features) that characterized the context-updating event (i.e., the previous trial). Therefore, even in the absence of any physical feature overlap across consecutive trials in a conflict task (say, moving from “RED in blue” to “GREEN in yellow”), the temporal context cue provided by an incongruent trial will facilitate the retrieval (or foster the maintenance) of the control state or task-set associated with that trial for a period of time, whose extent likely depends on the temporal statistics of the task environment (Egner et al., 2010). Note again that, as with the suggestion that internal control states can be bound into event files, the idea that a *temporal* context cue can determine the retrieval and temporary application of suitable processing strategies is not wedded to any particular view of what the exact trigger (e.g., conflict) or nature of the control state might be, and it naturally extends beyond the confines of the CSE. Thus, the same logic applies to all manner of local sequence effects, like enhanced response inhibition following stop trials in the stop-signal task (Bissett and Logan, 2012), or the proposal that subjects might adjust decision or response thresholds to resemble response times on a previous trial (Schmidt, 2013). These are all instances of the broader category of associations between temporal context and internal processing strategies.

### CREATURES OF HABIT: THE PAST IS (MOST OFTEN) THE FUTURE

Colloquially speaking, the role of temporal context cues as described above corresponds to fostering “expectations,” and the notion of the previous trial setting a local temporal context cue could be equated to the idea of “repetition expectancy” (Gratton et al., 1992; Egner, 2007); i.e., subjects, either explicitly or implicitly, expect forthcoming stimulation to resemble that of the recent past. The empirical literature on the role of expectations in the context of the CSE at first blush appears somewhat mixed, in part because the question of whether participants inherently expect successive trials to be similar is easily confused with the (orthogonal) question of whether manipulating expectations for congruent or incongruent trials can modulate congruency effects (e.g., Gratton et al., 1992; Aarts and Roelofs, 2011). Studies that manipulated expectancies by varying the relative probability of encountering different trial type transitions (congruency repetitions versus changes) have sometimes observed a modulation of the CSE by expectations (Duthoo et al., 2013, 2014b; Jimenez and Mendez, 2014) and sometimes not (Duthoo and Notebaert, 2012; Jimenez and Mendez, 2013, 2014). Importantly, though, a lack of expectation effects in these studies was essentially expressed as a failure to override the apparent default tendency to adapt to the previous trial type, i.e., of repetition expectancy (Duthoo and Notebaert, 2012; Jimenez and Mendez, 2013, 2014). In line with these findings, when participants are probed explicitly about their expectations of the forthcoming congruency, they exhibit a reliable bias toward expecting the present congruency type to be repeated (Duthoo et al., 2013; for equivalent findings in the domain of task-switching, see Duthoo et al., 2012). In sum, it can be argued that there is in fact consistent evidence for an inherent default setting whereby the organism expects a high degree of short-term autocorrelation (stability) in the environment—a *continuity bias*, which here is expressed in the form of an expectation for the congruency (or difficulty, etc.) of the next trial to resemble that of the previous one.

In line with the assumption of repetition expectancy, a pervasive bias for ongoing perception and decision-making to be strongly conditioned by (serially dependent on) the recent past has been well-documented by a number of elegant recent psychophysical studies (Cheadle et al., 2014; Fischer and Whitney, 2014). The idea that, on a moment-by-moment basis, we tend to (implicitly) assume that the immediate future resembles the immediate past, should in fact be unsurprising, as our cognitive apparatus is the result of evolutionary adaptation to a sensory world of high temporal autocorrelation: in the wild, what we see at one moment is a highly reliable predictor (combined with efference copy signals of eye-movements and other motor acts) of the visual input for the next moment (e.g., Dong and Atick, 1995). A particularly efficient way of capitalizing on this fact is to retrieve episodic memories that resemble the present situation, and which therefore allow us to predict the unfolding events ahead of time (for excellent, more extensive treatments of such a “memory-prediction” scheme, see Hawkins and Blakeslee, 2004; Clark, 2013). Anticipating our environment, through the use of episodic contextual cues, allows us to match, as closely as possible, previous experiences to incoming stimulation, and thus to optimize the nature and speed of our responses to our surroundings. As I highlighted above, this represents the shared purpose of low-level and high-level binding processes that link stimuli and contexts to suitable internal states and actions.

### SUMMARY

To summarize, the present proposal attempts to integrate distinct “associative” and “control-based” perspectives on the CSE (and related phenomena), by arguing that in the bigger picture these accounts can all be seen as describing complementary levels of learning with a shared goal; specifically, learning to link external stimulation to appropriate internal states (including appropriate action selection). At the most concrete level, this corresponds to associating physical stimulus features with specific motor responses; at a higher level of abstraction it involves binding contextual cues (including temporal frames) to internal attentional states and processing strategies (e.g., task sets). The encoding of an event into memory thus incorporates not only the associative binding of concrete stimulus features and actions, but also of concurrent internal states (including control settings), as well as more abstract external features that create a situational context (location, background, etc.), including an episodic context that places the present experience within a temporally extended reference frame. Translated back to the example of trial sequences in the Stroop task, participants continually encode (or rather, update) memories of event ensembles. As assumed by the feature integration and contingency learning perspectives, these ensembles include associations between concrete, physical stimulus and response features (e.g., “RED,” “blue ink,” “left index finger response”), as well as more abstract stimulus features (e.g., the categorization of the stimulus as “incongruent” or “difficult”). Importantly, these event ensembles furthermore incorporate contextual information, like stimulus location, both in the spatial and temporal reference frames, and all of these “external” event features become associated with the internal processing states that are engaged when dealing with the stimuli in question, like task- or conflict-specific top-down biasing strategies (e.g., “attend to color”)^3^. Retrieval of these internal states, along with the lower-level aspects of each episode file, is triggered by the encounter or prediction of event ensembles of (complete or partial) matching contextual cues, again ranging from physical stimulus characteristics to temporal context.

In this scheme, stimulus–response, categorical, and context-control associations can all occur simultaneously, though the degree to which any type of association is acquired and retrieved to drive task performance will naturally be determined in large part by the statistical structure of the task environment. An attractive proposal in this regard is that the cognitive apparatus is a “miser,” attempting to produce appropriate action while exerting the least mental effort possible (e.g., Botvinick, 2007; Kool et al., 2010). (This is of course also the point of forming stimulus–response and context-control links). The brain will therefore exploit correlational task structure any way it can (e.g., Melara and Algom, 2003): if we encounter cues that predict responses directly, we use them to bypass more complex and energetically expensive processing (e.g., in task designs that allow for contingency-learning), thus enhancing speed and saving effort. If stimulus–response learning is rendered impractical but there are cues that predict, for example, trial difficulty, then we use those cues to adapt top-down control settings to task demands. In other words, the acquisition and retrieval of context-control associations may represent something of a “last resort” for the organism (Bugg, 2014). In line with this proposition, it has been shown in proportion congruent manipulations in the Stroop task that when item-specific stimulus–response linkages are relatively frequent or salient, no “list-wide” effects of control are observed (Bugg et al., 2008; Blais and Bunge, 2010), but when direct stimulus–response learning is rendered less efficient, a list-wide control effect can be seen to emerge (Bugg and Chanani, 2011).

### OUTLOOK

More formal, empirical and simulation-based testing of the precise determinants of, and relationships between, the putative different levels of learning that I have proposed here will hopefully prove a fruitful endeavor for future investigations. Some particularly important lines of inquiry include the following: first, as emphasized throughout the paper, we do not presently know what exactly the more abstract trial properties or control processes are that are putatively being incorporated into episodic memory ensembles. Ongoing CSE research with experimental protocols that avoid low-level memory effects so far appears to point in the direction of a response-focused mechanism (Lee and Cho, 2013; Weissman et al., 2014), but the jury is still out on a definitive answer to this question. Secondly, the interplay between the different levels of learning advocated here is presently not well understood. In order to improve on this situation, researchers in this area will need to move from viewing low-level memory effects as “confounds” to incorporating independent manipulations of trial feature repetitions at different levels of abstraction, such that separate as well as potentially interactive contributions between, say, physical feature repetitions and control state repetitions can be assessed. A related question of great interest concerns the manner in which the binding of concrete and abstract episode features occurs at the level of neural mechanisms. The fast-paced (trial-by-trial) CSE should likely involve the type of quick episodic encoding (and retrieval) of trial features typically associated with medial temporal lobe (MTL) structures, including the hippocampus (e.g., Squire, 1992). An intriguing possibility, based on extant literature, is that as the binding process moves from more concrete (physical stimulus) features to more abstract (e.g., attentional state) features, the interaction between the MTL and cortical regions may shift along a posterior to anterior gradient—specifically, interactions with posterior ventral visual stream regions (and motor cortex) for concrete features (e.g., Kühn et al., 2011), the anterior temporal lobe stream for categorical stimulus features (e.g., Kensinger et al., 2003), and lateral prefrontal cortex and posterior parietal cortex in the binding of control-level features (e.g., Egner and Hirsch, 2005; King et al., 2012). A thorough examination of the mechanisms underlying multi-level learning effects that transcend the traditional separation of associative versus control-based cognitive processing should make for an exciting and highly important future avenue of research.

### Conflict of Interest Statement

The author declares that the research was conducted in the absence of any commercial or financial relationships that could be construed as a potential conflict of interest.

## Footnotes

1 Interestingly, there is in fact growing recent evidence that conflict (or its’ resolution) enhances long-term memory for task-relevant stimulus features (target stimuli; Krebs et al., 2013; Mayr et al., 2014; Rosner et al., 2014).

2 The findings of Jiang et al. (2014) also highlight the fact that observing additive effects of proportion congruent and previous trial congruency factors on performance does not necessarily imply that two independent processes are involved in bringing about these effects (e.g., Torres-Quesada et al., 2014). Both of these (additive) effects can be produced by a single mechanism that adapts its learning rate to the task environment.

3 This proposal is compatible with the possibility that other internal states will also become associated with the stimulus event, such as affective states, which may bring with them the acquisition of avoidance learning (e.g., Dreisbach and Fischer, 2012).
